# Real-World Evidence of Atezolizumab Efficacy as Part of First-Line Treatment for Extensive-Stage SCLC in Bulgaria

**DOI:** 10.3390/cancers18071129

**Published:** 2026-04-01

**Authors:** Manoela Manova, Boryana Ivanova, Rositsa Krasteva, Nikolay Conev, Tanya Zlatanova, Jeliazko Arabadjiev, Natalia Chilingirova, Mila Petrova, Assen Dudov, Bozhil Robev, Velko Minchev, Ivan Tonev, Krassimir Koynov, Daniel Penchev, Todor Georgiev, Antoan Rangelov, Alexandra Savova

**Affiliations:** 1 Department of Organization and Economy of Pharmacy, Faculty of Pharmacy, Medical University, 1000 Sofia, Bulgaria; 2 National Council on Prices and Reimbursement of Medicinal Products, 1431 Sofia, Bulgaria; 3 Medical Oncology Clinic, UniHospital, 4500 Panagyurishte, Bulgaria; 4 Medical Oncology Clinic, UMHAT St. Marina, 9010 Varna, Bulgaria; 5 Medical Oncology Clinic, Acibadem City Clinic UMHAT Tokuda, 1407 Sofia, Bulgaria; 6 Medical Oncology Clinic, Heart and Brain Hospital, 5800 Pleven, Bulgaria; 7 Medical Oncology Clinic, MHAT Nadezhda, 1330 Sofia, Bulgaria; 8 Medical Oncology Clinic, Acibadem City Clinic Mladost, 1784 Sofia, Bulgaria; 9 Medical Oncology Clinic, UMHAT “St. Ivan Rilski”, 1431 Sofia, Bulgaria; 10 Medical Oncology Clinic, SofiaMed University Hospital, 1797 Sofia, Bulgaria; 11 Complex Oncology Center, 4004 Plovdiv, Bulgaria; 12 Medical Oncology Clinic, MHAT Serdika, 1303 Sofia, Bulgaria; 13 Sqilline Health, 1766 Sofia, Bulgaria; 14 Faculty of Chemistry and Pharmacy, Sofia University “St. Kliment Ohridski”, 1164 Sofia, Bulgaria

**Keywords:** SCLC, real-world, atezolizumab, immune checkpoint inhibition, PD-L1, extensive-stage

## Abstract

**Background/Objectives**: Small cell lung cancer (SCLC) is a highly metastatic and lethal malignancy. Doublet chemotherapy consisting of a platinum compound and etoposide had remained the standard first-line regimen for decades, particularly for the majority of patients, who are diagnosed with extensive-stage disease. Over the past 5 years, results from the IMpower133 trial led to the approval of anti-PD-L1 agent atezolizumab as part of the first-line regimen for extensive-stage SCLC. The accumulation of real-world evidence on atezolizumab efficacy in this context is essential for corroborating trial results and further improving patient outcomes. Herein, we present real-world evidence from a Bulgarian cohort receiving atezolizumab as part of first-line extensive-stage SCLC treatment, comparing these data to the results of IMpower133. **Methods**: Data were extracted from the electronic health records of patients receiving atezolizumab plus chemotherapy as first-line treatment for extensive-stage SCLC in the period between 1 January 2022 and 31 December 2024. Analysis was conducted via the Danny Platform, an analytic platform for the integration of real-world evidence using embedded machine learning and LLM algorithms. Patients matching IMpower133 inclusion criteria were included in a group for comparison with trial results. **Results**: A total of 302 patients were included in the analysis. The real-world progression-free survival (PFS) in our study surpassed that in IMpower133 (7.6 versus 5.2 months), in addition to higher PFS rates beyond six months. A lower Objective Response Rate (ORR) (38.7% versus 65.4%) was noted in our real-world cohort, which we attribute to real-world documentation practices. **Conclusions**: Real-world evidence regarding PFS and treatment response generally align with the efficacy of adding atezolizumab to first-line extensive-stage SCLC treatment reported in the IMpower133 trial, while reflecting differences that may arise in routine clinical practice.

## Simple Summary

Small cell lung cancer accounts for a relatively small share of lung cancer cases, yet is highly lethal, without notable advances in treatment approaches over the past three decades. The recent introduction of immunotherapy as part of the initial chemotherapy regimens has contributed to improving survival, bringing a renewed hope for better patient outcomes. With the approval of atezolizumab for clinical use, however, it is essential to accumulate evidence on its efficacy and safety in real clinical practice. In the present study, we provide real-world evidence from Bulgarian patients receiving atezolizumab as part of their initial treatment for small cell lung cancer. The current data corroborate the notion that this newly introduced regimen delays disease progression to an extent comparable to that reported in the clinical trial that led to atezolizumab approval for small cell lung cancer.

## 1. Introduction

Lung cancer remains the leading cause of cancer-related mortality, causing an estimated 1.8 million deaths in 2022 alone [1]. Accounting for approximately 10–15% of lung cancer cases, small-cell lung cancer (SCLC) is an aggressive and lethal high-grade carcinoma of mostly neuroendocrine origin [2]. In numbers, this amounts to over 250.000 new cases of SCLC and an estimated 200.000 deaths globally each year [1]. Among common solid tumor types, SCLC has a particularly high mortality rate that has seen little improvement over the past two decades [3]. Nearly all patients diagnosed with the disease are current or former heavy smokers [4]. As one of the malignancies with the strongest link to tobacco consumption, SCLC prevalence mirrors tobacco consumption trends [5]. Smoking cessation programs have thus contributed to a decrease in SCLC incidence in countries such as the US, UK, and Australia, while low- and middle-income countries have seen a rise in cases [6,7].

SCLC is characterized by early and frequent metastasis, and oligometastatic disease is common at diagnosis. In line with this metastatic predilection, patients with SCLC exhibit the highest numbers of circulating tumor cells among those affected by solid tumors [8]. As much as 80% of patients present with stage IV disease at diagnosis [2]. For the two-thirds of patients that present with extensive-stage SCLC at diagnosis, meaning that a single tolerable radiotherapy field can no longer encompass the tumor spread, a systemic doublet chemotherapy regimen of a platinum agent and etoposide has remained the standard first-line option for decades [9,10]. According to the American Cancer Society, extensive-stage SCLC describes cancers that have spread widely throughout the lung, to the other lung, or to other parts of the body, including the bone marrow. While cytotoxic therapy elicits potent responses in treatment-naïve SCLC (response rates of 60–65%), these are transient, and chemoresistance ensues, leading to survival durations of approximately 1 year for those with an extensive stage disease [11]. A median survival of just 7 months was reported based on US registry data for the 1983–2012 period [3]. A dismal prognosis, repeated failure to improve treatment outcomes, and low sample availability had limited SCLC funding and research for nearly four decades [9].

The frequent autoimmune paraneoplastic syndromes observed in patients with SCLC, occurring at the highest rate among all tumor types, highlight its immunogenic potential [12]. Further, the high mutational burden and correspondingly high number of neoantigens further incentivized the utilization of immune checkpoint inhibitors for the treatment of SCLC [13,14,15,16]. Atezolizumab is a humanized monoclonal antibody against programmed death ligand-1 (PD-L1), which blocks its interaction with programmed death (PD-1) to reinvigorate anti-tumor T-cell responses [17]. Atezolizumab monotherapy exhibited a favorable safety profile, with acceptable side effects and a sustained response in a subset of patients with relapsed or refractory SCLC [18]. Importantly, adding atezolizumab to first-line chemotherapy significantly prolonged overall survival (OS) and progression-free survival (PFS) when compared to chemotherapy alone [19]. The encouraging results of the IMpower133 study (https://clinicaltrials.gov/study/NCT02763579 accessed on 2 October 2025), which described the first significant improvement in OS and PFS in more than two decades, led to the approval of atezolizumab in combination with carboplatin and etoposide as first-line treatment for adults with extensive-stage SCLC [9]. The IMpower133 is a phase 3 double-blind, placebo-controlled study in 403 previously untreated patients who received carboplatin–etoposide with either atezolizumab (201 patients) or placebo (202 patients). Adults were considered eligible if they had histologically or cytologically confirmed extensive-stage SCLC, measurable disease per RECIST v1.1, an ECOG performance status of 0–1, and no prior systemic therapy for extensive-stage disease. Patients with previously treated asymptomatic central nervous system metastases were allowed. The main exclusion criteria were a history of autoimmune disease or prior exposure to CD137 agonists or immune checkpoint inhibitors. Treatment consisted of an induction cycle followed by maintenance atezolizumab or placebo until progression or unacceptable toxicity, with progression-free survival as primary endpoint [19]. Subsequent analysis of IMpower133 data indicated that favorable responses occurred independently of biomarker status [20]. Successful trial results led to the approval of multiple immune checkpoint inhibitors in 2019–2020, two of which had their FDA approvals withdrawn shortly after [9].

While invaluable, trial insights have been constrained by the inclusion and exclusion trial criteria, which are likely conducive to favorable patient outcomes. Thus, whether anti-PD-L1 inhibitors possess intrinsic superiority in the context of SCLC remains to be conclusively substantiated in the real-world setting [9]. It is therefore critical to obtain further insight into the external validity of trial results in real clinical practice, which would inform their rational application [21,22]. At present, the body of real-world evidence regarding atezolizumab efficacy in SCLC is growing, yet remains limited.

Herein, we provide real-world data from Bulgarian patients receiving atezolizumab plus platinum-based doublet chemotherapy as first-line treatment for extensive-stage SCLC, collected over a three-year period. The obtained real-world data allowed for a comparison to the IMpower133 trial, corroborating its major conclusions and providing a basis for further comparative analyses.

## 2. Materials and Methods

### 2.1. Patient Data

Real-world patient data for this study was retrospectively extracted from electronic health records (EHRs) across 48 hospitals (university, multispecialty, and oncology hospitals) in Bulgaria. The data were extracted from structured and unstructured text through the implementation of LLM algorithms. Data analysis was conducted via the Danny Platform (Sqilline Health, Sofia, Bulgaria; https://sqilline.com/ accessed on 20 October 2025), an analytic platform that integrates large amounts of real-world data from EHRs using embedded machine learning and large language model (LLM) algorithms to facilitate the extraction of information from free text in different languages. For this report, the data was further manually checked for consistency, errors, and missing values, wherever applicable.

### 2.2. End Points

Primary endpoints included PFS, objective response rate (ORR), and clinical benefit rate (CBR). PFS was defined as living with the disease for a certain period, but without disease progression. ORR was defined as the proportion of patients achieving a complete or partial response to treatment for a given period. CBR was defined as the proportion of patients achieving a complete response, partial response, or stable disease over a given period. The treatment response was evaluated according to the RECIST v1.1. The 95% confidence intervals for ORR and CBR were calculated as per the Wilson score method for binomial variables.

### 2.3. Survival Analysis

Survival outcomes were visualized via Kaplan–Meier curves, with confidence intervals calculated using Greenwood’s method, and differences between the real-world cohort and the Impower133 trial were assessed using the log-rank test. In comparison between RWD and clinical trial treatment outcomes, one of the challenges is that the underlying baseline characteristics between the two groups can be different. This can result in a bias in the comparison and hence interpretation if these different baselines are not taken into account. For instance, a difference in the PFS may not be the result of a difference in drug effectiveness but rather due to an older patient group in the real-world. To account for such differences, we applied iterative proportional fitting (IPF) to balance a set of chosen characteristics prior to our survival analyses. For a given characteristic, IPF works by deriving weights to adjust the underlying distributions in order to match a target distribution. For instance, if an RCT cohort consists of 50% men and 50% women but the real-world data has a different proportion (60% vs. 40%), then IPF will derive weights in such a way (e.g., more weights for the female patients) that the weighted sum would result in 50:50 as seen in the RCT. The characteristics considered included Eastern Cooperative Oncology Group (ECOG) performance status, sex, age, smoking status, inactive brain metastases at baseline as well as previous chemotherapy, radiotherapy, and surgery.

## 3. Results

### 3.1. Patient Eligibility

Eligibility for the real-world cohort was aligned with the main inclusion and exclusion criteria of the Impower133 trial. A total of 302 patients with extensive-stage SCLC received the combination of atezolizumab with carboplatin and etoposide between 1 January 2022 and 31 December 2024. Of these, 167 (55.3%) received the combination as a first-line regimen. These were designated as the Initial Treatment group and were eligible for subsequent analysis. In Bulgaria, histological confirmation through biopsy is standard clinical practice for the diagnosis of lung cancer, including SCLC, and is routinely performed. All patients included in this group had a verified diagnosis of extensive-stage SCLC, confirmed histologically, with immunohistochemical (IHC) investigation performed on the biopsy specimens. The rest (44.7%, *n* = 135) were considered non-eligible for comparisons with IMpower133 data and were correspondingly designated as the Non-eligible group. For further comparison, a small subset of eight patients with inactive brain metastases at baseline was added to the Initial Treatment group. This group included a total of 175 patients and is referred to as the Initial Treatment with Inactive BM hereafter. The criteria for inactive brain metastases are the same as per the Impower133 study. Inactive brain metastases were defined as previously treated and asymptomatic brain lesions without radiologic evidence of progression at treatment initiation. Their status was confirmed from the available electronic health records, including the treating oncologist’s conclusions based on CT or MRI performed in routine clinical practice before treatment start. Patient grouping is summarized in Table 1 below, and in the Supplementary Materials—Figure S1 patient flow diagram is provided for the compared cohorts. Hereafter, all percentages (except for those describing the proportion of previous regimens) are presented according to the total number of patients in the given group or based on the number of patients for whom the data in question were available.

### 3.2. Previous and Subsequent Regimens

Only three patients (1.8%) of the Initial Treatment group had received a previous regimen, namely, carboplatin plus etoposide. Prior regimens had been used by two patients from the Non-eligible group, with one receiving atezolizumab plus carboplatin and the other cisplatin plus etoposide (0.7% *n* = 1 each). In the Impower133 study 8 patients received previous treatments. More than half of the Initial Treatment group patients (60.4%, *n* = 101) went on to receive subsequent regimens versus a comparable proportion (66.6%, *n* = 90) of the Non-eligible group. Subsequent treatment regimens are summarized in Table 2. Of note, some patients had multiple subsequent regimens, leading to a total of 144 regimens (for 90 patients) in the Non-eligible group and 160 regimens in the Initial treatment group, in which 101 patients from the total 167 received a subsequent treatment directly after atezolizumab + carboplatin + etoposide. Within the Initial Treatment group, atezolizumab monotherapy (53.8%, *n* = 86) and topotecan (13.8%, *n* = 22) were most common, followed by cyclophosphamide plus epirubicin (10.0%, *n* = 16), among other, less common options (Table 2). Maintenance atezolizumab (*n* = 76, 52.8%) and topotecan monotherapy (*n* = 20, 13.9%) were also the most common. Similar patterns were observed for both groups in terms of the subsequent regimen received directly after atezolizumab plus carboplatin and etoposide (Table 3), and in the Supplementary Materials—Table S3: Subsequent regimens directly after atezolizumab and carboplatin plus etoposide in the Initial Treatment group and Table S4: Subsequent regimens directly after atezolizumab and carboplatin plus etoposide in the Non-eligible group are provided for the compared cohorts. A number of less common regimens were noted in the Non-eligible group, which included microtubule-stabilizing agent paclitaxel, immunosuppressants methotrexate and mitoxantrone, and durvalumab, another anti-PD-L1 immune checkpoint inhibitor (Table 2), and in the Supplementary Materials—Table S1: Subsequent regimens in the Initial Treatment group (*n* = 101 patients) and Table S2: Subsequent regimens in the Non-eligible group (*n* = 90 patients) are provided for the compared cohorts. In the Initial Treatment group, 17 patients (10.2%) did not proceed to maintenance with atezolizumab: 16 due to disease progression or brain metastasis and one due to drug unavailability. In the Non-eligible group, one patient had to transition to cisplatin-based combination therapy due to carboplatin unavailability.

### 3.3. Comparison Between Real-World Evidence and IMpower133

#### 3.3.1. Baseline Characteristics

The baseline characteristics of the Non-eligible, Initial Treatment, Initial Treatment with Inactive BM, and IMpower133 groups are summarized in Table 4. The comparison presented hereafter focuses on the Initial Treatment and IMpower133 cohorts, highlighting deviations in the Non-eligible and Initial Treatment with Inactive BM, if such are present.

The distribution of sexes was comparable between the Initial Treatment and trial cohorts, with men accounting for 70.1% in the former versus 64.2% in the latter group. The median age in the Initial Treatment group was slightly higher, at 66 years, compared to 64 years in the IMpower133 cohort. That is, most patients (*n* = 96, 57.5%) of the Initial Treatment group were over the age of 65, which was not the case in IMpower133 (*n* = 90, 44.8%). As per trial inclusion criteria, all patients in both cohorts had an ECOG performance status of 0–1, yet ECOG 1 was more common in the real-world cohort, accounting for 80.8% (*n* = 135), versus 63.7% (*n* = 128) among IMpower133 cases. The observed difference in ECOG distribution suggests that in real-world settings, patients are more likely to initiate therapy under a condition of mild functional impairment.

Among the 74 Initial Treatment group patients for whom smoking data were available, current smokers were predominant (79.7%, *n* = 59), as opposed to the IMpower133 cohort, where the majority of patients were former (58.7%, *n* = 118), and 36.8% were current smokers (*n* = 74). A slightly higher proportion (25.9%, *n* = 23) of Non-eligible patients were former smokers relative to Initial Treatment group patients (17.6%, *n* = 13). Never-smokers were a minority in either cohort (2.7%, *n* = 2 in Initial Treatment versus 4.5%, *n* = 9 in IMpower133). In line with inclusion criteria, previous anticancer treatments were not common in the Initial Treatment and IMpower133 cohorts, being slightly more frequent in the latter (Table 4). Of note, a considerable proportion of patients in the Non-eligible group (31.1%, *n* = 42) had undergone radiotherapy. The Non-eligible group also exhibited the highest proportion of patients with brain metastasis at enrolment (25.2%, *n* = 34), followed by the IMpower133 cohort (8.5%, *n* = 17) and the Initial Treatment with Inactive BM group (4.6%, *n* = 8).

#### 3.3.2. Treatment Responses and Survival Analysis

Treatment response data were available for 111 patients in the Initial Treatment group and 185 patients in the IMpower133 cohort, as summarized in Table 5. A considerable difference in ORR was noted between the real-world and trial setting, reported in 38.7% (95% CI 29.6–48.5, *n* = 43) of Initial Treatment group patients and 65.4% (95% CI 58.0–72.2, *n* = 121) of IMpower133 participants. The ORR in the Non-eligible group was also low relative to that among trial participants, at 40.6% (95% CI: 31.1–50.9). Meanwhile, the CBR was largely comparable between the Initial Treatment and IMpower133 groups, at 82.9% (95% CI 74.6–89.3, *n* = 92) versus 88.1% (95% CI 82.5–92.4, *n* = 163), with no statistically significant difference between groups (chi-square *p* = 0.207).

**Figure 1:**
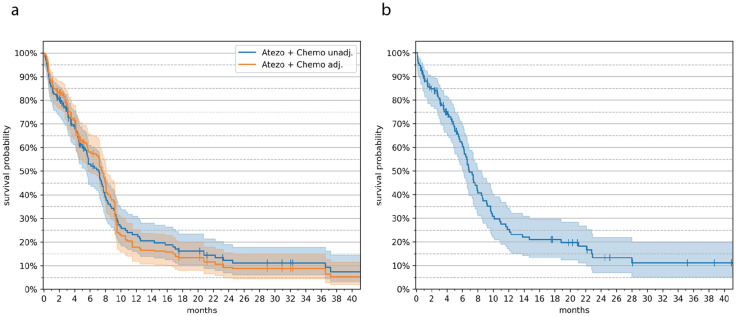
Progression-free survival probability curves from real-world extensive-stage SCLC data. (**a**) Progression-free survival probability in the Initial Treatment group. The curves shown are before (blue) and after (orange) adjustment via iterative proportional fitting. (**b**) Progression-free survival probability in the Non-eligible group.

A longer PFS was reported in the Initial Treatment Group (7.6 [95% CI 5.8–8.1] months); when compared to the IMpower133 trial (5.2 [95% CI 4.4–5.6]), this difference was statistically significant by log-rank test (*p* = 0.0279). A smaller proportion of patients from the Initial Treatment group (71.2%, *n* = 119) experienced progression-related events when compared to IMpower133 participants (85.1%, *n* = 171). Survival probabilities in the Initial Treatment and Non-eligible groups are summarized in Figure 1. While the PFS rate was initially lower in the Initial Treatment group, this trend was reversed from the fourth month onward, with 58% (95% CI 50–66) versus 30.9% (95% CI 24.3–37.5) (*n* = 58) at 6 months and 18% (95% CI 12–25) versus 12.6% (95% CI 7.9–17.4) at 12 months (Table 6). Of note, the Initial Treatment group exhibited a considerably higher proportion of patients without progression at 20 months, that is, 10.8% (*n* = 18) versus a single patient (0.5%) in the IMpower133 cohort. The PFS rate at 20 months was 13% in the Initial Treatment group versus ~5% in the trial. PFS trends in the Non-eligible and Initial Treatment with Inactive BM groups were largely consistent with that in the Initial Treatment group, outperforming IMpower133 data (Table 6).

## 4. Discussion

The present study assessed the real-world efficacy of atezolizumab in combination with carboplatin plus etoposide for the treatment of extensive-stage SCLC, adding valuable insights to a limited body of evidence from real clinical practice. Corroborating the results from the IMpower133 clinical trial is essential in order to confirm the intrinsic efficacy of PD-L1 checkpoint inhibition in conjunction with doublet chemotherapy in this disease context. We report a longer PFS among patients in the real-world setting when compared to IMpower133 results.

In terms of baseline demographic characteristics, our real-world group effectively matched the IMpower133 cohort, with any potential confounders accounted for via IPF. The median age as well as the proportion of patients above and below 65 years of age were comparable to those of an earlier real-world study of atezolizumab efficacy against extensive-stage SCLC, which was conducted in Canada [23]. The study in question reported a median PFS of 6.0 months, which was lower than that observed in our real-world cohort (7.6 months). It should be noted, however, that the Canadian study was not limited to an ECOG performance status of 0–1, with a quarter of atezolizumab-treated patients having an ECOG performance status ≥ 2. The J-TAIL-2 study, which included an IMpower133-like subgroup in terms of inclusion and exclusion criteria, reported a median PFS of 5.4 months, compared to median PFS of 4.8 months in the IMpower133-unlike subgroup, confirming a more favorable outcome among patients that are selected in accordance with trial criteria (e.g., an ECOG status of 0–1) [24]. Meanwhile, the ORR (69.1% [59.6–77.6]) reported in the Japanese IMpower133-like group was consistent with IMpower133 results (65.4% [58.0–72.2]) and thus considerably higher than reported in our study (38.7% [29.6–48.5]). Finally, the proportion of patients receiving subsequent treatment in the IMpower133-like subgroup of the J-TAIL-2 study was comparable to that in our Initial Treatment group (62.7% vs. 60.4%), while also comparable to that in the IMpower133-unlike subgroup (61.6%). Careful comparisons of subsequent regimens and their efficacy are essential, as the inevitable chemoresistance regardless of the specific regimen employed remains a major challenge in SCLC, contributing to exponential attrition rates after each subsequent therapy line [25].

Real-world evidence on atezolizumab in combination with carboplatin plus etoposide from South Korea reported a median PFS of 6.0 months, in addition to an ORR in line with IMpower133 results and higher than reported herein [26]. Recent studies from Greece and Spain assessing chemoimmunotherapy outcomes in extensive-stage SCLC reported a median PFS approaching that in our Initial Treatment group, at 6.5 and 6.8 months, respectively [27,28]. Similar studies from the UK, France, and China reported lower median PFS values in the 5.2–5.8-month range [29,30,31]. Meanwhile, a median PFS of 4.5 months was reported in an Indian cohort, hinting at potential differences between high- and low-resource settings [32]. Caution should be exercised when comparing our current data to these previously published studies, as their study populations exhibit a consistent rate of brain metastasis at enrolment > 20%, which is considerably greater than the 4.6% for the Initial Treatment with Inactive BM group in our study (4.6%) and IMpower133 (8.5%). This matter is further complicated by the need for detailed information regarding brain metastases, i.e., whether they are stable previously treated metastases or active metastases that are yet to be treated. As much as a quarter of patients with SCLC present with brain metastases at diagnosis, with up to an additional 50% developing brain metastases during the disease course [33]. A real-world study of atezolizumab for extensive-stage SCLC in Italy subdivided patients based on the number of atezolizumab cycles received, both as part of combination therapy and as maintenance. A greater number of atezolizumab cycles was associated with a longer PFS, ranging from 39.2 months in those having undergone >31 cycles to 6.9 months in the <10 cycles group [34].

While complete and partial responses were rare and most common, respectively, in previously published real-world reports as well as in our work, a difference can be noted in the higher rate of stable disease in our Initial Treatment group (44.1%) when compared to both IMpower133 and other reports (<25%) [26,30]. This relates to the low ORR we report relative to the trial and subsequent studies. That is, real-world documentation practices often lead to patients being recorded as having stable disease, which results in a lower ORR, while contributing to the CBR, which was in line with that of IMpower133 (82.9% vs. 88.1%). While patients with SCLC who experience durable responses are extremely rare, case reports highlight sustained disease control, as described for a 76-year-old former smoker [35]. Efforts are being focused on the analysis of long-term survivor cohorts in a bid to identify robust predictors of a sustained response [36].

In the present study, there was a striking difference in the proportion of former smokers when compared to the IMpower133 cohort, where they constituted a majority of the patients (17.6% versus 58.7%). Nearly all SCLC patients are or have been tobacco smokers, and the duration as well as intensity of smoking have been shown to influence relative risk [37]. Thus, the above-described discrepancy can be interpreted as rather circumstantial, as both current and former smokers fall within the ever-smoker category. The prevalence of tobacco smoking remains high in Balkan countries, including Bulgaria, when compared to that across western and northern Europe [38].

To our knowledge, this is the first study to report real-world data on the use of atezolizumab as part of first-line extensive-stage SCLC regimens in Bulgaria. While it provides valuable insights into the real-world efficacy of atezolizumab in combination with carboplatin and etoposide as first-line therapy, certain limitations should be acknowledged. The retrospective design of this study introduces inherent methodological limitations, including potential selection bias, information bias, and incomplete clinical documentation typical of real-world datasets. The absence of a true control group, similar to that in the IMpower133 trial, limits the ability to draw definitive causal conclusions regarding the benefit of introducing atezolizumab into first-line doublet chemotherapy regimens. A detailed analysis of OS data from our real-world cohort would further strengthen the understanding of treatment effectiveness in this population. Finally, an aspect that remains to be addressed in this real-world cohort is the safety of atezolizumab in combination with carboplatin and etoposide, as safety outcomes were not systematically analysed in the present study.

## 5. Conclusions

The present study adds to existing real-world evidence on the efficacy of atezolizumab in combination with first-line carboplatin and etoposide for the treatment of extensive-stage SCLC. The real-world cohort was associated with a median PFS of 7.3 months, compared to the median PFS of 5.3 months reported in the RCT, highlighting the potential added value of real-world data for contextualising and complementing trial outcomes. Our results support the efficacy reported in the IMpower133 trial, providing a basis for further comparisons and a careful evaluation of the intrinsic efficacy of PD-L1 blockade via atezolizumab in this context. 

## Figures and Tables

**Table 1:** Study groups employed from comparison.

Group	Number of Patients
Initial Treatment	167
Initial Treatment with Inactive BM	175
Non-eligible	135
IMpower133	201

**Table 2:** Subsequent regimens in the Non-eligible (*n* = 90) and Initial Treatment (*n* = 101 patients) groups.

Regimen	Non-Eligible (*n* = 144 Regimens)	Initial Treatment (*n* = 160 Regimens)
Atezolizumab	76 (52.8%)	86 (53.8%)
Topotecan	20 (13.9%)	22 (13.8%)
Cyclophosphamide + epirubicin (pharmorubicin)	15 (10.4%)	16 (10.0%)
Carboplatin + etoposide	11 (7.6%)	12 (7.5%)
Cisplatin + irinotecan	6 (4.2%)	7 (4.4%)
Carboplatin + irinotecan	5 (3.5%)	2 (1.2%)
Cisplatin + etoposide	2 (1.4%)	5 (3.1%)
Irinotecan	2 (1.4%)	3 (1.9%)
Atezolizumab + carboplatin + etoposide	--	2 (1.2%)
Cyclophosphamide + epirubicin (pharmorubicin) + vincristine	--	2 (1.2%)
Atezolizumab + cisplatin + etoposide	1 (0.7%)	--
Atezolizumab + cyclophosphamide	1 (0.7%)	--
Carboplatin + durvalumab + etoposide	1 (0.7%)	--
Cisplatin + paclitaxel	1 (0.7%)	--
Cyclophosphamide + methotrexate + mitoxantrone	1 (0.7%)	--
Durvalumab	1 (0.7%)	--
Ifosfamide	1 (0.7%)	3 (1.9%)

**Table 3:** Subsequent regimens directly after atezolizumab and carboplatin plus etoposide in the Non-eligible and Initial Treatment groups.

Regimen	Non-Eligible (*n* = 135 Regimens)	Initial Treatment (*n* = 167 Regimens)
Atezolizumab	74 (54.8%)	84 (50.3%)
Topotecan	6 (4.4%)	10 (6.0%)
Cyclophosphamide + epirubicin (pharmorubicin)	4 (3.0%)	3 (1.8%)
Carboplatin + etoposide	2 (1.5%)	--
Carboplatin + irinotecan	2 (1.5%)	--
Atezolizumab + cisplatin + etoposide	1 (0.7%)	--
Cisplatin + irinotecan	1 (0.7%)	3 (1.8%)
Cyclophosphamide + epirubicin (pharmorubicin) + vincristine	--	1 (0.6%)

**Table 4:** Demographic characteristics of the real-world evidence patient groups and the IMpower133 cohort.

Characteristic	Values	Non-Eligible	Initial Treatment	IMpower133
Age (at start)		*n* = 135	*n* = 167; 175	*n* = 201
	Median	66	66; 66	63
	Mean ± SD	65.3 ± 8.3	64.6 ± 8.7; 64.6 ± 8.6	-
	Q1–Q3	60–72	59–71; 59–71	-
	Min–Max	42–86	37–80; 37–80	28–90
Age group		*n* = 135	*n* = 167; 175	*n* = 201
	<65	62 (45.9%)	71 (42.5%); 74 (42.3%)	111 (55.2%)
	≥65	73 (54.1%)	96 (57.5%); 101 (57.7%)	90 (44.8%)
Sex		*n* = 135	*n* = 167; 175	*n* = 201
	M	95 (70.4%)	117 (70.1%); 122 (69.7%)	129 (64.2%)
	F	40 (29.6%)	50 (29.9%); 53 (30.3%)	72 (35.8%)
ECOG		*n* = 135	*n* = 167; 175	*n* = 201
	0	31 (23.0%)	32 (19.2%); 32 (18.3%)	73 (36.3%)
	1	104 (77.0%)	135 (80.8%); 143 (81.7%)	128 (63.7%)
Smoking status		*n* = 89	*n* = 74; 80	*n* = 201
	Never smoked	1 (1.1%)	2 (2.7%); 2 (2.5%)	9 (4.5%)
	Current smoker	65 (73.0%)	59 (79.7%); 64 (80%)	74 (36.8%)
	Former smoker	23 (25.9%)	13 (17.6%); 14 (17.5%)	118 (58.7%)
Metastases		*n* = 135	*n* = 167; 175	*n* = 201
	Brain metastases at enrollment	34 (25.2%)	0 (0.0%); 8 (4.6%)	17 (8.5%)
Previous anticancer treatments		*n* = 135	*n* = 167; 175	*n* = 201
	Chemotherapy	2 (1.5%)	3 (1.8%); 3 (1.7%)	8 (4.0%)
	Radiotherapy	42 (31.1%)	14 (8.4%); 18 (10.3%)	25 (12.4%)
	Cancer-related surgery	7 (5.2%)	14 (8.4%); 16 (9.1%)	33 (16.4%)

Initial Treatment values include the groups with and without patients with inactive BMs, separated by a semicolon (;).

**Table 5:** Treatment responses across real-world and IMpower133 groups.

	Non-Eligible	Initial Treatment	Impower133
Best Response	*n* = 91	*n* = 111; 116	*n* = 185
Complete response	1 (1.1%)	6 (5.4%); 6 (5.2%)	5 (2.7%)
Partial response	36 (39.6%)	37 (33.3%); 39 (33.6%)	116 (62.7%)
Stable disease	47 (51.6%)	49 (44.1%); 52 (44.8%)	42 (22.7%)
Progression	7 (7.7%)	19 (17.2%); 19 (16.2%)	22 (11.9%)
	*n* = 91	*n* = 111; 116	*n* = 185
ORR	37 (40.6%, 95% CI: 31.1–50.9)	43 (38.7%, 95% CI: 29.6–48.5); 38.8 (30.4–47.8)	121 (65.4%, 95% CI: 58.0–72.2)
CBR	84 (92.3%, 95% CI: 84.9–96.2%)	92 (82.9%, 95% CI: 74.6–89.3); 83.6 (75.8–89.2)	163 (88.1%, 95% CI: 82.5–92.4%)

Initial Treatment values include the groups with and without patients with inactive BMs, separated by a semicolon (;).

**Table 6:** Progression-free survival data for the real-world and IMpower133 groups.

	Non-Eligible (*n* = 135)	Initial Treatment (*n* = 167)	Initial Treatment with Inactive BM (*n* = 175)	IMpower133 (*n* = 201)
# of events	87 (64.4%)	119 (71.2%)	126 (72.0%)	171 (85.1%)
Median	6.9 months (6.1–8.4)	7.6 months (5.8–8.1)	7.3 months (6.1–8.0)	5.2 months (4.4–5.6)
Patients at risk; PFS rate				
4 months	74 (54.8%); 75% (66–82)	95 (56.9%); 71% (64–78)	100 (57.1%); 72% (64–78)	147 (73%); ~81%
6 months	57 (42.2%); 61% (52–70)	63 (37.7%); 58% (50–66)	68 (38.9%); 58% (50–66)	58 (28.9%) 30.9% (24.3–37.5)
8 months	37 (27.4%); 41% (31–50)	46 (27.5%); 45% (37–53)	47 (26.9%); 42% (33–50)	41 (20.4%); ~22%
12 months	23 (17.0%); 25% (17–30)	27 (16.2%); 18% (12–25)	28 (16%); 17% (11–24)	21 (10.4%); 12.6% (7.9–17.4)
16 months	19 (14.1%); 21% (13–30)	22 (13.2%); 16% (10–23)	23 (13.1%); 15% (10–22)	3 (1.4%); ~11%
20 months	15 (11.1%); 20% (12–28)	18 (10.8%); 13% (8–20)	19 (10.9%); 13% (8–19)	1 (0.5%) ~5%

The PFS rate is presented as a percentage (95% CI).

## Data Availability

Data supporting reported results can be provided by the corresponding authors upon reasonable request.

## Supplementary Materials

The following supporting information can be downloaded at: https://www.mdpi.com/article/10.3390/cancers18071129/s1, Figure S1: Patient flow diagram; Table S1: Subsequent regimens in the Initial Treatment group (*n* = 101 patients); Table S2: Subsequent regimens in the Non-eligible group (*n* = 90 patients); Table S3: Subsequent regimens directly after atezolizumab and carboplatin plus etoposide in the Initial Treatment group; Table S4: Subsequent regimens directly after atezolizumab and carboplatin plus etoposide in the Non-eligible group.

## Abbreviations

The following abbreviations are used in this manuscript:

PFS	Progression-free survival
SCLC	Small cell lung cancer
OS	Overall survival
ORR	Objective response rate
CBR	Clinical benefit rate
