# Supplementary material for: Real-World Evidence of Atezolizumab Efficacy as Part of First-Line Treatment for Extensive-Stage SCLC in Bulgaria
Source: Cancers (Basel). 2026 Apr 1;18(7):1129. doi: 10.3390/cancers18071129 (PMC13072253; doi:10.3390/cancers18071129)
Supplement: Supplementary file 1 [file cancers-18-01129-s001.zip › Supplementary Tables.pdf]

Table S1. Subsequent regimens in the Initial Treatment (n = 101 patients) group.

Regimen	Initial treatment (n = 160 regimens)
Atezolizumab	86 (53.8%)
Topotecan	22 (13.8%)
Cyclophosphamide + epirubicin (pharmorubicin)	16 (10.0%)
Carboplatin + etoposide	12 (7.5%)
Cisplatin + irinotecan	7 (4.4%)
Cisplatin + etoposide	5 (3.1%)
Ifosfamide	3 (1.9%)
Irinotecan	3 (1.9%)
Carboplatin + irinotecan	2 (1.2%)
Atezolizumab + carboplatin + etoposide	2 (1.2%)
Cyclophosphamide + epirubicin (pharmorubicin) + vincristine	2 (1.2%)

Table S2. Subsequent regimens in the Non-eligible (n = 90 patients) group.

Regimen	Non-eligible (n = 144 regimens)
Atezolizumab	76 (52.8%)
Topotecan	20 (13.9%)
Cyclophosphamide + epirubicin (pharmorubicin)	15 (10.4%)
Carboplatin + etoposide	11 (7.6%)
Cisplatin + irinotecan	6 (4.2%)
Carboplatin + irinotecan	5 (3.5%)
Cisplatin + etoposide	2 (1.4%)
Irinotecan	2 (1.4%)
Atezolizumab + cisplatin + etoposide	1 (0.7%)
Atezolizumab + cyclophosphamide	1 (0.7%)
Carboplatin + durvalumab + etoposide	1 (0.7%)
Cisplatin + paclitaxel	1 (0.7%)
Cyclophosphamide + methotrexate + mitoxantrone	1 (0.7%)
Durvalumab	1 (0.7%)
Ifosfamide	1 (0.7%)

Table S3. Subsequent regimens directly after atezolizumab and carboplatin plus etoposide in the Initial Treatment group.

Regimen	Initial treatment (n = 167 regimens)
Atezolizumab	84 (50.3%)
Topotecan	10 (6.0%)
Cyclophosphamide + epirubicin (pharmorubicin)	3 (1.8%)
Cisplatin + irinotecan	3 (1.8%)

Cyclophosphamide + epirubicin (pharmorubicin) + vincristine	1 (0.6%)

Table S4. Subsequent regimens directly after atezolizumab and carboplatin plus etoposide in the Non-eligible group.

Regimen	Non-eligible (n = 135 regimens)
Atezolizumab	74 (54.8%)
Topotecan	6 (4.4%)
Cyclophosphamide + epirubicin (pharmorubicin)	4 (3.0%)
Carboplatin + etoposide	2 (1.5%)
Carboplatin + irinotecan	2 (1.5%)
Atezolizumab + cisplatin + etoposide	1 (0.7%)
Cisplatin + irinotecan	1 (0.7%)
