# Supplementary figures and images for: Real-World Evidence of Atezolizumab Efficacy as Part of First-Line Treatment for Extensive-Stage SCLC in Bulgaria
Source: Cancers (Basel). 2026 Apr 1;18(7):1129. doi: 10.3390/cancers18071129 (PMC13072253; doi:10.3390/cancers18071129)

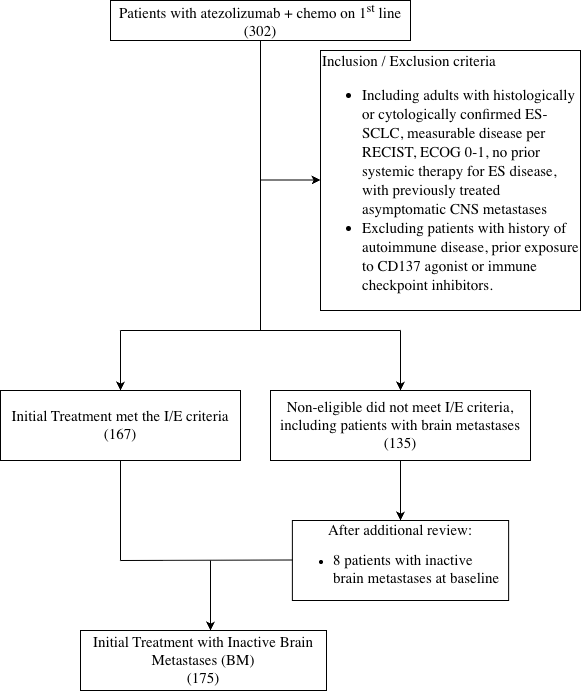

Supplement: Supplementary file 1 [file cancers-18-01129-s001.zip › Supplementary Figure S1.png]
